# Self-Perception of Dental Esthetics among Dental Students at King Saud University and Their Desired Treatment

**DOI:** 10.1155/2021/6671112

**Published:** 2021-02-22

**Authors:** Aminah M. El Mourad, Ahoud Al Shamrani, Maha Al Mohaimeed, Sarah Al Sougi, Shahad Al Ghanem, Wassan Al Manie

**Affiliations:** ^1^Division of Operative Dentistry, Department of Restorative Dental Sciences, College of Dentistry, King Saud University, P.O. Box 5967, Riyadh 11432, Saudi Arabia; ^2^College of Dentistry, King Saud University, P.O. Box 5967, Riyadh 11432, Saudi Arabia

## Abstract

**Objective:**

Dental esthetic procedures are popular worldwide, and dentists are responsible for recommending several treatment options to their patients. To do this competently, dentists must have an educated opinion of their features. Therefore, we aimed to evaluate the self-perception of dental esthetics among male and female dental students and assess its effect on their desired treatment. *Methodology*. We conducted a questionnaire-based cross-sectional study involving 450 male and female dental students from five academic years with different grade point averages (GPAs) and monthly household incomes. The questionnaire comprised four sections. The subjects selected their teeth-color preferences using a commercial shade guide. The outcomes were presented using descriptive statistics and were compared with Pearson's chi-square test. The level of statistical significance was set at a *p*-value of 0.05.

**Results:**

Female students (52%) showed a significantly higher preference for changing their tooth color (shade B1 was desired most often), whereas male students reported a higher need for orthodontic treatment and ceramic veneers (*p* < 0.0001 and *p*=0.002). Fifth-year students were more satisfied with their teeth color as compared with 1st-year students (*p*=0.047). High-GPA students showed significantly lower confidence regarding their smiles (*p*=0.030). A high percentage of students (39.1%) with household incomes of less than 10,000 SR preferred tooth-colored restorations.

**Conclusion:**

We concluded that the majority of dental students were confident about their smiles. Senior students were more satisfied with their teeth color, while younger students desired whiter teeth. Bleaching and orthodontics were the most desired treatment options.

## 1. Introduction

Modern society is placing increasing value on physical esthetics, with dental esthetic procedures gaining prominence for their ability to complement facial beauty [1, 2]. The positive psychosocial effect of pleasant facial esthetics is a major reason for patients (especially young individuals) to seek corrective dental treatment [3, 4]. The need for esthetic facial changes is often self-perceived and can influence an individual's self-confidence and quality of life [5–9].

Among the many dental principles on facial proportion, several are directed toward anterior teeth since they are critical for a pleasant smile [10, 11]. In addition to smile design, esthetic dentistry has other aspects, such as facial symmetry, occlusion, the role of the buccal corridor, the “golden proportion,” and appearance of the gingiva––all of which positively or negatively influence a patient's smile, confidence, and beauty [12–17]. The perception of beauty is distinct for each individual; this subjectivity might influence their degree of concern toward esthetics, depending on their gender, age, ethnicity, socioeconomic status, marital status, level of education, occupation, familial influence, cultural exposure, and social media [18–22].

Esthetic treatments are designed to combine several dental specialties, including restorative, orthodontic, prosthodontic, and surgical treatments [23]. Therefore, dental students, who are exposed to all branches of dentistry, should be educated about the available esthetic treatments and their respective indications; this reflects the competency of a new dentist [2].

The dental esthetic requirements of the general population have been studied in detail, and dental students worldwide have also been surveyed [24, 25]. It is important that dental students have adequate knowledge and awareness of the differences in how dental professionals and laypeople perceive dental esthetics; this may ultimately help them to better understand their patients' needs and expectations and formulate optimal treatment plans. However, only few studies have been conducted on dental students' perception of their esthetics and desired treatment in Saudi Arabia [23, 26, 27]. Furthermore, no Saudi Arabian studies have compared the self-perception of their own dental esthetics among male and female dental students with respect to factors such as their academic year, GPA, and monthly household income. To address this research gap, we aimed to evaluate self-perceived esthetics among male and female dental students from different academic years at our university and assess the effect of their perception on the desired esthetic treatment.

## 2. Materials and Methods

A cross-sectional study was conducted among 450 male and female dental students aged 17–23 years at King Saud University, Riyadh, Saudi Arabia. First, second, third, fourth, and fifth-year dental students were recruited; 45 male and 45 female students were included from each academic year. The institutional review board of our university approved the study protocol (approval number: E-18-2808), and informed consent was obtained from each participant.

Data were collected using structured questionnaires that were randomly distributed among the students. These questionnaires were prepared in English and translated to Arabic for easier understanding. The questions were based on a previous study with modifications [18]. The questionnaire comprised a covering page that described the project and confirmed the subjects' confidentiality, followed by four sections: section A comprised the gender, current academic year, grade point average (GPA) range of the previous academic year, and monthly household income range of the student (Table 1); section B comprised “yes/no” questions that included 15 items pertaining to self-perception of dental esthetics (Tables 2 and 3) [18]; section C comprised multiple-answer questions on the desired esthetic treatments (Table 4) [23]; and section D allowed the students to select their preferred tooth shade using the BlueLine shade guide (Ivoclar Vivadent, Schaan, Liechtenstein, Germany) (Table 5) [23]. We included the most commonly used shades in clinical practice: A1, A2, A3, B1, B2, B3, and BL1 [28]. The shade codes were hidden and numbered randomly to ensure that the students' knowledge of the shades did not affect their selection [29]. A pilot experiment was conducted at the beginning of the study by distributing the questionnaire to eight students to ensure its clarity and comprehensibility.

Data were analyzed using the SPSS 21.0 software (IBM Inc., Chicago, USA). Subject characteristics and outcome variables were presented using descriptive statistics (frequencies, percentages, mean, and standard deviation). Pearson's chi-square test was used to compare the distribution of categorical responses in relation to the categorical study variables. The level of statistical significance was set at a *p*-value of 0.05.

## 3. Results

Of the 450 total subjects, 385 responded to the questionnaire (response rate = 85.5%). Gender was approximately equally distributed: 51.9% of subjects were females, and 48.1% were males. We intended to enroll an equal number of subjects from each academic year, but the number of responses from the 4th- and 5th-year students was marginally less. Approximately 70.1% of subjects had a GPA of 4.5–5, and 64.4% had a monthly household income of over 20,000 SAR (Table 1).

Among the 15 questions on the self-perception of esthetics, the question “Is there someone you believe has a better smile than you?” received the highest number of “yes” responses (88.6%). We recorded valid “no” responses for the following questions: “Are you satisfied with the way your gums look?” (21.3%); “Are you self-confident about smiling?” (20.5%); “Do you like the way your teeth are shaped?” (19.5%). The remaining 11 questions had “yes” responses ranging from 17.9 to 70.6% (Table 2). We compared these responses to the characteristics of the subjects (gender, academic year, GPA, and monthly household income) and recorded the following results.

### 3.1. Gender

We observed a statistically significant difference between males and females for “yes” responses to three questions: “Do you ever put your hand over your mouth when you smile?”; “Do you photograph better from one side of your face? (e.g., preference for taking a selfie from the right or left side)”; “Are you satisfied with the way your gums look?” For these three questions, a higher proportion of female subjects responded “yes” (31%, 60%, and 83.5% respectively) as compared with the male subjects (19.5%, 46.5%, and 73.5%, respectively); this was statistically significant (*p*=0.009, *p*=0.008, and *p*=0.017, respectively) (Table 3).

Gender significantly influenced the students' selection of desired esthetic treatment. “Orthodontic treatment and ceramic veneers” were selected by a higher number of male subjects (50.8% and 14.1%, respectively) as compared to female subjects (32.5% and 5%, respectively); this was statistically significant (*p* < 0.0001 and *p*=0.002) (Table 4). In terms of shade preference, a higher percentage of female subjects (52%) chose “B1 shade” among all the other shades, as compared with the male students' preference for “B1” (35.7%); this was statistically significant (*p* < 0.0001) (Table 5).

### 3.2. Academic Year

A higher number of senior students (3rd, 4th, and 5th academic years) than younger students (1st and 2nd academic years) responded “yes” to the following two questions: “Do you feel you have enough knowledge of the available esthetic treatment options?” and “Do you prefer to have tooth-colored restorations on your posterior teeth knowing that amalgam and gold restorations have higher survival rates?” This observation was statistically significant (*p* < 0.0001 and *p* < 0.0001) (Table 4).

A higher number of 5th-year students responded “yes” to the question “Are you satisfied with your teeth color?” as compared with the 1st-, 2nd-, 3rd-, and 4th-year students; this was statistically significant (*p*=0.047) (Table 3). The 5th-year students' advanced academic level, knowledge, and clinical experience may have influenced the difference in shade selection. A majority of the 1st-year students preferred the “BL1” shade (Table 5).

### 3.3. GPA

In terms of the students' GPA, a statistically significant difference was observed for one question: “Are you self-confident about smiling?” A higher proportion of subjects with GPAs of 3.75–4.49 and < 3.75 responded “yes” (88% and 85.7%, respectively), compared to the proportion (75.9%) of subjects with a GPA of 4.5–5.0; this was statistically significant (*p*=0.030) (Table 3).

### 3.4. Monthly Household Income

The subjects' responses regarding the choice of tooth-colored restorations were significantly different across the four categories of monthly household income, wherein 39.1% and 31.6% of subjects with a monthly household income of <10,000 SR and 10,000–15,000 SR, respectively, responded “yes” as compared with subjects from the other two categories (15,000–20,000 and > 20,000) of monthly household income (17.1% and 17.3%, respectively) (*p*=0.019) (Table 4).

## 4. Discussion

Having pleasing facial features is essential for an individual to form their esthetic perception and value their physical attractiveness [30]. Eyes, followed by the smile, are the most prominent features in a person's facial appearance [31]. The self-perceived image of dental esthetics can influence a person's self-confidence, satisfaction, quality of life, psychological condition, and social interactions [32–39]. Moreover, evaluating a patient's dental esthetic perception is crucial for dentists to understand their needs and manage their expectations regarding the treatment outcomes [40].

In this study, we observed that 79.5% of male and female respondents were confident about their smiles, with no statistically significant difference with respect to gender. This result is in accordance with those of previously published articles [15, 18, 23]. Women are more aware of and concerned about their dental esthetics as compared with men [22, 40–44]; we observed a similar phenomenon where most of our female subjects frequently covered their mouths with their hands and preferred to be photographed from the “good” side of their face. This may be because females pay more attention to their dental appearance than males and tend to be more critical when assessing their smile esthetics [40]. Most of our subjects were satisfied with the appearance of their gums, with females being more satisfied as compared to males. This finding is consistent with that of a previous study where the majority of female dental students were satisfied with their gums [23].

Dental students with a GPA of 4.5–5.0 reported lower levels of satisfaction with their smiles. This might be because students who perform well academically might undergo a more rigorous critical appraisal regarding their smile esthetics [15].

Esthetic color perception is subjective and depends on an individual's gender, experience, social circumstances, and educational background [31]. Several previous studies have compared tooth-color esthetic perception between laypersons and dental practitioners [18, 30, 40, 41, 43]. In our study, we observed that 1st-year students preferred “BL1” to whiten their teeth. This might be because the media emphasizes extremely white teeth as attractive and aspirational for most people [23]. The 1st-year students' opinions were closer to those of laypersons due to their insufficient knowledge and experience in analyzing a smile correctly and professionally [41]. In contrast, older students were more realistic and preferred the appearance of natural teeth, which resulted in them showing more satisfaction regarding their current tooth color [31, 45]; this was particularly true for 5th-year students. This finding is in line with that of another study, wherein a low percentage of 5th-year students were found to desire whiter teeth [15]. In terms of a gender-based shade preference, we observed that a significantly higher percentage of females chose the “B1” shade as compared with male subjects. On the other hand, Ansari et al. [46] reported that male subjects were more interested in procuring the whitest shade of teeth as compared to the female subjects.

We found that the level of academic experience (academic year in dental school) directly correlated with the students' knowledge of treatment options. This result is similar to that of a previous study, where treatment knowledge was related to awareness of facial attractiveness, higher education level, and more clinical experience [23].

In terms of desired dental treatment, we recorded that 66.5% of our subjects preferred posterior tooth-colored restorations despite knowing that amalgam and gold restorations have higher survival rates. Another study showed similar results; it demonstrated that 69.2% of dental students preferred composite restorations over amalgam in posterior teeth, indicating that dental students place a high level of importance on the appearance of their teeth [23, 27].

Teeth whitening was the most desired treatment, followed by orthodontic treatment. This can be attributed to the students' desire to conform to an “attractive” image of straight and white teeth since they are more sensitive than laypersons in detecting deviations from the “ideal” dental appearance [41]. These findings are similar to the results reported in the study of Subait et al. [27].

Orthodontics was the most desired treatment among 4th-year students. Male students demonstrated a higher need for orthodontic treatment and ceramic veneers. The demand for orthodontic treatment has been increasing among adolescents and young adults due to increased concerns about appearance and esthetics, which makes it one of the most desired treatments in the general population [47–52]. On the contrary, da Silva et al. [18] found that most dental students in their study were satisfied with the shape and alignment of their teeth; this finding was attributed to the fact that most students had undergone orthodontic treatment before their enrollment in dental school.

Lastly, we observed that students with a household income of less than 10,000 SR had a greater preference for tooth-colored restorations. This is because low-income populations would tend to seek affordable yet esthetic dental treatment.

A limitation in this study was the use of only one of the available approaches for assessing dental esthetic perception, as there has not yet been a consensus regarding an optimal, standardized, and objective evaluation method. Future investigations should consider the use of objective clinical examinations to evaluate students' perception of their smile esthetics.

## 5. Conclusions

The majority of dental students in our study were confident about their smiles. Females were more aware and concerned about their dental esthetics than males. Younger students preferred whiter teeth; however, older students in higher academic years tended to be more satisfied with the natural appearance of their teeth. The most desired treatment options were teeth whitening and orthodontics.

## Figures and Tables

**Table 1:** Characteristics of the subjects (*n* = 385).

Characteristics	Number (%)
*Gender*	
Male	185 (48.1)
Female	200 (51.9)

*Academic year*	
1st	80 (20.8)
2nd	80 (20.8)
3rd	80 (20.8)
4th	70 (18.2)
5th	75 (19.5)

*GPA*	
4.5–5	270 (70.1)
3.75–4.49	108 (28.1)
<3.75	7 (1.8)

*Monthly household income (in SAR)*	
<10,000	23 (6.0)
10,000–15,000	38 (9.9)
15,000–20,000	76 (19.7)
Above 20,000	248 (64.4)

GPA, grade point average.

**Table 2:** Subjects' responses regarding self-perception of dental esthetics.

Questions regarding self-perception	Response		Total
	Yes (%)	No (%)	
Are you confident about smiling?^*∗*^	306 (79.5)	79 (20.5)	385
Do you ever put your hand over your mouth when you smile?	98 (25.5)	287 (74.5)	385
Do you photograph better from one side of your face? (e.g., take a selfie from the right or left side)	206 (53.5)	179 (46.5)	385
Is there someone you believe has a better smile than you?	341 (88.6)	44 (11.4)	385
Do you look at magazines and wish you had a smile like the models?	185 (48.1)	200 (51.9)	385
When you read a fashion magazine, are your eyes drawn to the model's smile?	272 (70.6)	113 (29.4)	385
When you look at your smile in the mirror, do you see any defects in your teeth or gums?	229 (59.5)	156 (40.5)	385
Are you satisfied with your teeth color?	197 (51.2)	188 (48.8)	385
Are you satisfied with the way your gums look?^*∗*^	303 (78.7)	82 (21.3)	385
Do you show too many or too few teeth when you smile?	139 (36.1)	246 (63.9)	385
Do you show too much or too little gum when you smile?	108 (28.1)	277 (71.9)	385
Are your teeth too long or too short?	75 (19.5)	310 (80.5)	385
Are your teeth too wide or too narrow?	69 (17.9)	316 (82.1)	385
Are your teeth too square or too round?	69 (17.9)	316 (82.1)	385
Do you like the way your teeth are shaped?^*∗*^	310 (80.5)	75 (19.5)	385

^*∗*^A response of “no” was considered valid.

**Table 3:** Comparison of the subjects' responses toward desired esthetic treatments with respect to their gender, academic year, and GPA (part 1).

Questions regarding self-perception	Gender-number (%)		*p*-value	Academic year-number (%)					*p*-value	GPA-number (%)			*p*-value
	Male	Female		1st	2nd	3rd	4th	5th		4.5–5	3.75–4.49	<3.75	
Are you confident about smiling? (Yes)	140 (75.5)	166 (83.0)	0.075	57 (71.3)	62 (77.5)	66 (82.5)	61 (87.1)	60 (80.0)	0.165	205 (75.9)	95 (88.0)	6 (85.7)	0.030^*∗*^

Do you ever put your hand over your mouth when you smile? (Yes)	36 (19.5)	62 (31.0)	0.009^*∗*^	23 (28.8)	20 (25.0)	22 (27.5)	13 (18.6)	20 (26.7)	0.654	77 (28.5)	20 (18.5)	1 (14.3)	0.104

Do you photograph better from one side of your face? (e.g., take a selfie from the right or left side) (yes)	86 (46.5)	120 (60.0)	0.008^*∗*^	37 (46.3)	36 (45.0)	54 (67.5)	38 (54.3)	41 (54.7)	0.035^*∗*^	145 (53.7)	58 (53.7)	3 (42.9)	0.825

Is there someone you believe has a better smile than you? (Yes)	169 (91.4)	172 (86.0)	0.099	74 (92.5)	71 (88.8)	71 (88.8)	63 (90.0)	62 (82.7)	0.413	239 (88.5)	96 (88.9)	6 (85.7)	0.967

Do you look at magazines and wish you had a smile like the models? (Yes)	94 (50.8)	91 (45.5)	0.297	41 (51.3)	36 (45.0)	45 (56.3)	28 (40.0)	35 (46.7)	0.324	131 (48.5)	50 (46.3)	4 (57.1)	0.823

When you read a fashion magazine, are your eyes drawn to the model's smile? (Yes)	131 (70.8)	141 (70.5)	0.947	51 (63.8)	48 (60.0)	64 (80.0)	54 (77.1)	55 (73.3)	0.024^*∗*^	187 (69.3)	80 (74.1)	5 (71.4)	0.649

When you look at your smile in the mirror, do you see any defects in your teeth or gums? (Yes)	112 (60.5)	117 (58.5)	0.684	49 (61.3)	45 (56.3)	42 (52.5)	47 (67.1)	46 (61.3)	0.423	160 (59.3)	64 (59.3)	5 (71.4)	0.810

Are you satisfied with your teeth color? (Yes)	91 (49.2)	106 (53.0)	0.455	35 (43.8)	38 (47.5)	40 (50.0)	34 (48.6)	50 (66.7)	0.047^*∗*^	133 (49.3)	59 (54.6)	5 (71.4)	0.357

Are you satisfied with the way your gums look? (Yes)	136 (73.5)	167 (83.5)	0.017^*∗*^	60 (75.0)	69 (86.3)	64 (80.0)	57 (81.4)	53 (70.7)	0.155	220 (81.5)	78 (72.2)	5 (71.4)	0.124

Do you show too many or too few teeth when you smile? (Yes)	72 (38.9)	67 (33.5)	0.269	38 (47.5)	27 (33.8)	24 (30.0)	26 (37.1)	24 (32.0)	0.161	93 (34.4)	43 (39.8)	3 (42.9)	0.575

Do you show too much or too little gum when you smile? (Yes)	52 (28.1)	56 (28.0)	0.981	31 (38.8)	19 (23.8)	14 (17.5)	19 (27.1)	25 (33.3)	0.030^*∗*^	75 (27.8)	33 (30.6)	0	0.215

Are your teeth too long or too short? (Yes)	39 (21.1)	36 (18.0)	0.446	16 (20.0)	14 (17.5)	19 (23.8)	13 (18.6)	13 (17.3)	0.844	52 (19.3)	21 (19.4)	2 (28.6)	0.828

Are your teeth too wide or too narrow? (Yes)	33 (17.8)	36 (18.0)	0.967	14 (17.5)	17 (21.3)	11 (13.8)	12 (17.1)	15 (20.0)	0.771	45 (16.7)	23 (21.3)	1 (14.3)	0.552

Are your teeth too square or too round? (Yes)	30 (16.2)	39 (19.5)	0.401	14 (17.5)	11 (13.8)	15 (18.8)	16 (22.9)	13 (17.3)	0.704	47 (17.4)	20 (18.5)	2 (28.6)	0.736

Do you like the way your teeth are shaped? (Yes)	143 (77.3)	167 (83.5)	0.125	61 (76.3)	66 (82.5)	61 (76.3)	62 (88.6)	60 (80.0)	0.291	216 (80.0)	88 (81.5)	6 (85.7)	0.891

^*∗*^Statistically significant.

**Table 4:** Comparison of the subjects' responses toward desired esthetic treatments with respect to their gender, academic year, GPA, and monthly household income (part 2).

Questions regarding treatment	Do you feel you have enough knowledge of the available esthetic treatment options?	Do you prefer to have tooth-colored restorations on your posterior teeth knowing that amalgam and gold restorations have higher survival rates? (Yes)	Your desired esthetic dental treatment (multiple responses)						
			Teeth whitening	Orthodontic treatment	Tooth-colored restorations	Ceramic veneers	Crowns	Partial dentures or implants	I Do not need any treatment
*Response*									
Yes	284 (73.8)	256 (66.5)	251 (65.2)	159 (41.3)	77 (20)	36 (9.4)	22 (5.7)	11 (2.9)	66 (17.1)
No	101 (26.2)	129 (33.5)	134 (34.8)	226 (58.7)	308 (80)	349 (90.6)	363 (94.3)	374 (97.1)	319 (82.9)

*Gender-Number (%) (yes)*									
Male	133 (71.9)	117 (63.2)	114 (61.6)	94 (50.8)	44 (23.8)	26 (14.1)	15 (8.1)	8 (4.3)	31 (16.8)
Female	151 (75.5)	139 (69.5)	137 (68.5)	65 (32.5)	33 (16.5)	10 (5.0)	7 (3.5)	3 (1.5)	35 (17.5)
*p*-value	0.421	0.194	0.157	<0.0001^*∗*^	0.074	0.002^*∗*^	0.052	0.097	0.847

*Academic year-Number (%) (yes)*									
1st	47 (58.8)	37 (46.3)	52 (65.0)	26 (32.5)	12 (15.0)	10 (12.5)	4 (5.0)	4 (5.0)	12 (15.0)
2nd	48 (60.0)	50 (62.5)	54 (67.5)	31 (38.8)	17 (21.3)	8 (10.0)	7 (8.8)	5 (6.3)	11 (13.8)
3rd	68 (85.0)	64 (80.0)	51 (63.8)	33 (41.3)	17 (21.3)	5 (6.3)	2 (2.5)	1 (1.3)	13 (16.3)
4th	53 (75.7)	48 (68.6)	51 (72.9)	40 (57.1)	19 (27.1)	7 (10.0)	6 (8.6)	1 (1.4)	9 (12.9)
5th	68 (90.7)	57 (76.0)	43 (57.3)	29 (38.7)	12 (16.0)	6 (8.0)	3 (4.0)	0	21 (28.0)
*p*-value	<0.0001^*∗*^	<0.0001^*∗*^	0.391	0.037^*∗*^	0.356	0.721	—	—	0.089

*GPA-Number (%)*									
4.5–5	196 (72.6)	176 (65.2)	181 (67.0)	103 (38.1)	45 (16.7)	26 (9.6)	14 (5.2)	9 (3.3)	47 (17.4)
3.75–4.49	83 (76.9)	74 (68.5)	67 (62.0)	52 (48.1)	28 (25.9)	9 (8.3)	7 (6.5)	2 (1.9)	18 (16.7)
<3.75	5 (71.4)	6 (85.7)	3 (42.9)	4 (57.1)	4 (57.1)	1 (14.3)	1 (14.3)	0	1 (14.3)
*p*-value	0.690	0.457	0.299	0.141	0.006^*∗*^	0.836	0.545	—	0.965

*Monthly household income-number (%)*									
<10,000	15 (65.2)	14 (60.9)	14 (60.9)	10 (43.5)	9 (39.1)	2 (8.7)	1 (4.3)	1 (4.3)	3 (13.0)
10,000–15000	22 (57.9)	27 (71.1)	19 (50.0)	21 (55.3)	12 (31.6)	3 (7.9)	5 (13.2)	2 (5.3)	6 (15.8)
15,000–20,000	57 (75.0)	51 (67.1)	52 (68.4)	28 (36.8)	13 (17.1)	8 (10.5)	3 (3.9)	1 (1.3)	13 (17.1)
>20,000	190 (76.6)	164 (66.1)	166 (66.9)	100 (40.3)	43 (17.3)	23 (9.3)	13 (5.2)	7 (2.8)	44 (17.7)
*p*-*value*	0.075	0.871	0.192	0.281	0.019^*∗*^	0.972	—	—	0.944

^*∗*^Statistically significant; (yes) if the question answer was yes.

**Table 5:** Comparison of the subjects' responses regarding shade preference for teeth with respect to their characteristics.

Characteristics	Type of color							*p*-value
	None	B3	B1	A2	B2	A1	BL1	
*Gender*								
Male	53 (28.6)	8 (4.3)	66 (35.7)	5 (2.7)	12 (6.5)	5 (2.7)	36 (19.5)	<0.0001^*∗∗*^
Female	51 (25.5)	0	104 (52.0)	1 (0.5)	6 (3.0)	12 (6.0)	26 (13.0)	

*Academic year*								
1st	18 (22.5)	0	24 (30.0)	0	1 (1.3)	2 (2.5)	35 (43.8)	—
2nd	11 (13.8)	0	46 (57.5)	0	4 (5.0)	1 (1.3)	18 (22.5)	
3rd	22 (27.5)	0	44 (55.0)	1 (1.3)	6 (7.5)	2 (2.5)	5 (6.3)	
4th	24 (34.3)	0	28 (40.0)	5 (7.1)	1 (1.4)	10 (14.3)	2 (2.9)	
5th	29 (38.7)	8 (10.7)	28 (37.3)	0	6 (8.0)	2 (2.7)	2 (2.7)	

*GPA*								
4.5–5	64 (23.7)	2(0.7)	130 (48.1)	2 (0.7)	10 (3.7)	12 (4.4)	50 (18.5)	—
3.75–4.49	36 (33.3)	5(4.6)	38 (35.2)	4 (3.7)	8 (7.4)	5 (4.6)	12 (11.1)	
<3.75	4 (57.1)	1(14.3)	2 (28.6)	0	0	0	0	

*Monthly household income (in SAR)*								
<10,000	6 (26.1)	0	12 (52.2)	1 (4.3)	0	3 (13.0)	1 (4.3)	—
10,000–15,000	13 (34.2)	0	15 (39.5)	0	1 (2.6)	0	9 (23.7)	
15,000–20,000	21 (27.6)	1 (1.3)	33 (43.4)	1 (1.3)	5 (6.6)	1 (1.3)	14 (18.4)	
Above 20,000	64 (25.8)	7 (2.8)	110 (44.4)	4 (1.6)	12 (4.8)	13 (5.2)	38 (15.3)	

^*∗*^Statistically significant. GPA: Grade point average.

## Data Availability

Due to the ethical and legal responsibility to respect participants' rights to privacy and to protect their identity, the clinical dataset is not publicly available.

## Additional Points

*Clinical Significance.* The concept of an ideal smile differs for dental professionals and laypersons. We recommend that this difference be considered each time a dentist designs a treatment plan since patients may often have exaggerated expectations of treatment outcomes (e.g., having unnaturally white and straight teeth). Understanding this difference in perception will help dental practitioners recognize their patients' needs, manage their expectations, participate in their decision-making process, and design effective treatment plans to produce better outcomes.
